# Silent Speech Eyewear Interface: Silent Speech Recognition Method Using Eyewear and an Ear-Mounted Microphone with Infrared Distance Sensors †

**DOI:** 10.3390/s24227368

**Published:** 2024-11-19

**Authors:** Yuya Igarashi, Kyosuke Futami, Kazuya Murao

**Affiliations:** 1 Graduate School of Information Science and Engineering, Ritsumeikan University, 2-150 Iwakuracho, Ibarakishi 567-8570, Japan; yuya.igarashi@iis.ise.ritsumei.ac.jp (Y.I.); murao@fc.ritsumei.ac.jp (K.M.); 2 Digital Spirit Teck, Kusatsu 525-8577, Japan

**Keywords:** silent speech interaction, skin motion sensing, eyewear, infrared distance sensor, hands-free input

## Abstract

As eyewear devices such as smart glasses become more common, it is important to provide input methods that can be used at all times for such situations and people. Silent speech interaction (SSI) has the potential to be useful as a hands-free input method for various situations and people, including those who have difficulty with voiced speech. However, previous methods have involved sensor devices that are difficult to use anytime and anywhere. We propose a method for SSI that involves using an eyewear device equipped with infrared distance sensors. The proposed method measures facial skin movements associated with speech from the infrared distance sensor mounted on an eyewear device and recognizes silent speech commands by applying machine learning to time series sensor data. The proposed method was applied to a prototype system including a sensor device consisting of eyewear and ear-mounted microphones to measure the movements of the cheek, jaw joint, and jaw. Evaluations 1 and 2 showed that five speech commands could be recognized with an F value of 0.90 and ten longer speech commands with an F value of 0.83. Evaluation 3 showed how the recognition accuracy changes with the combination of sensor points. Evaluation 4 examined whether the proposed method can be used for a larger number of speech commands with 21 commands by using deep learning LSTM and a combination of DTW and kNN. Evaluation 5 examined the recognition accuracy in some situations affecting recognition accuracy such as re-attaching devices and walking. These results show the feasibility of the proposed method for a simple hands-free input interface, such as with media players and voice assistants. Our study provides the first wearable sensing method that can easily apply SSI functions to eyewear devices.

## 1. Introduction

The utilization of speech interaction as a prevalent hands-free input method in mobile, desktop, wearable devices, and smart speakers has become widespread. Nevertheless, this method comes with drawbacks, including the potential to attract attention from nearby individuals [1], causing a disturbance, and making private speech content accessible to outsiders. Additionally, speech-recognition accuracy tends to decline in environments with substantial background noise [2]. Consequently, employing speech interaction in public places becomes challenging.

To tackle these challenges, there has been active research into silent speech interaction (SSI). SSI involves utilizing non-voiced speech as a means of speech interaction, showing potential as a hands-free input method in various scenarios and as an assistive technology for individuals with dysphonia (e.g., congenital or acquired disorders of the pharynx or vocal cords, e.g., due to old age). SSI inherits the advantages of speech interaction, such as not disrupting the user’s activities and eliminating the need to learn new operation commands since it leverages speech ability [2]. Moreover, the speech content of SSI is highly secure [3], making it inconspicuous in public places and avoiding the attention of those nearby.

Earlier research endeavors proposed various methods, including the recognition of lip movements based on a frontal camera image [4,5] and the identification of oral-muscle activity and mouth movements through sensors, such as acceleration/angular velocity sensors [2] and electromyography [6], applied to the skin of the face. Other techniques involved sensors on the palate and tongue (EPG [7,8] and PMA [9]), ultrasound echo imaging [10], and the attachment of sensors (e.g., strain sensor [11,12], acceleration sensor [13]) to a mask. However, these methods have not always been an acceptable style for constant use anytime and anywhere in a wearable environment due to having to attach sensors to the skin of the face, hold a sensor, place the camera in front of the face, place a sensor in the mouth, and cover the face with a mask.

The popularity of eyewear devices, including smart glasses designed for augmented reality/XR/virtual reality and for listening to music, is on the rise. Ear-mounted microphones and headsets are already widely adopted and socially acceptable. Despite their popularity, there have been limited proposals for implementing SSI exclusively using these devices. The integration of SSI functions into these devices could prove valuable for input operations while using eyewear devices. Additionally, it could extend SSI capabilities to situations and individuals for whom previous methods may not be suitable.

We propose a method of recognizing silent speech commands using a sensor device consisting of eyewear and ear-mounted microphones with infrared distance sensors. The proposed method recognizes silent speech commands by attaching the infrared distance sensors to the device to measure the skin movement of the face (e.g., cheeks, jaw joint, jaw) in conjunction with the mouth during speech. We constructed a prototype system. Five evaluations were conducted. In Evaluation 1, it was confirmed that the proposed method was feasible as a simple hands-free input interface. In Evaluation 2, we considered the combination patterns of multiple devices and examined the necessary sensor components. In Evaluation 3, we evaluated whether the proposed method can be used for long-time speech commands when using a voice assistant. In Evaluation 4, we examined whether estimation is possible using multiple analysis methods for a larger number of speech commands. In Evaluation 5, we investigated whether the system could be used in some situations affecting recognition accuracy such as re-attaching devices and walking.

Note that we published the concept of the proposed method in a short paper at the International Symposium on Wearable Computers (ISWC 2022) [14]. This paper added Section 7 (Evaluation 4) and Section 8 (Evaluation 5). This is the first study to provide a wearable sensing method that can easily apply SSI functions to eyewear devices.

## 2. Related Research

### 2.1. Silent Speech Interaction

SSI has many of the advantages of speech recognition, such as the ability to use the user’s speech, eliminating the need to learn new gesture commands when compared with gesture input. In addition, since SSI can be used even by those who have difficulty with vocalization for some reason, various methods have been proposed and studied. Methods based on camera images and ultrasound echo images include a method for estimating speech commands by acquiring lip images of a speaker using a cell phone camera [4,5] and a method for detecting a user’s silent speech using a handheld ultrasound image sensor [10]. However, these methods require the user to hold a device such as a camera in front of his/her face, which constrains the user’s hands. Methods that use inaudible speech include methods that capture faint vocal cord vibrations [15,16], methods that recognize mumbled voices from a microphone attached to the user’s skin [16], and methods that record speech by capturing inaudible sounds of breathing with a microphone [17]. However, the user must speak to accurately recognize the content of speech with these methods, which may allow people in the vicinity to overhear the user’s voice. Methods that place sensors in the mouth include the method using electropalatography (EPG), in which capacitance sensors are placed in the mouth to recognize the contact pattern between the palate and tongue [7,8,16,18,19]. In the method using permanent magnet articulography (PMA), a magnet is attached to the lips or mouth (tongue) [9]. However, these cannot be easily used by people for whom it is undesirable to have a sensor in the mouth. Methods to attach sensors directly to the face include attaching an acceleration/angular velocity sensor to the throat to measure skin movements accompanying mouth movements [2], and using electromyography to measure muscle movements around the mouth [6]. However, these methods are not suitable for some people who do not want to wear sensors on their skin, or because they are conspicuous. Methods using mask-type devices include a method that measures the displacement of the facial mask during a speech by using the mask containing an accelerometer [13] and a strain sensor (printed strain sensor [11], a flexible and highly sensitive strain sensor [12]). However, these are not suitable for situations where the mouth is not to be covered or for people who do not like to wear masks (e.g., people who have difficulty breathing). Studies on the social acceptability of SSI [3] have shown that SSI can protect private information even in public places because of its silent speech and that it is easily accepted by users because it is less conspicuous to others, even if it is less accurate than voiced interactions. Compared with these previous methods, this study aims to develop a method to easily use SSI only with popular wearable devices such as eyewear and ear microphone devices.

### 2.2. Sensing with Infrared Distance Sensors

Previous studies have used infrared distance sensors to measure skin movements. Some methods use earable devices. For example, there are ear accessory devices to recognize facial expressions gestures [20] and earphones to recognize gestures of the direction in which the ear is pulled [21]. Some methods use eyeglasses. For example, there is a method for recognizing smiles [22], a method for recognizing eight facial expressions in daily life [23], a gesture input method for face rubbing [24], and methods for recognizing eye activities, such as gaze movement and gaze direction [25,26,27]. Some methods use a head-mounted display (HMD). For example, there is a method to recognize touch gestures based on cheek skin movement [28] and a method for mapping one’s facial expressions to those of an avatar in virtual space [29,30] and a method for mapping one’s facial movements to a computer graphics model for use in animation production [30]. Other examples include a mouthpiece to recognize tongue gestures [31], a wristband to recognize hand-shape gestures [32], a ring to recognize finger movement gestures [33], and a mask to recognize facial expressions of the mouth [34]. Previous studies have shown that infrared distance sensors can measure skin movements. In addition, infrared distance sensors are suitable for wearable devices that are continuously used because of their light weight, small size, and little use of data for low power consumption. Therefore, an infrared distance sensor is suitable for the proposed method.

### 2.3. Mouth Recognition Methods

Various methods have been used to recognize the state of the mouth by measuring the face and head with sensors. A method to monitor snacking in daily life by measuring the movement of temporal muscles with a pressure sensor installed in a cap [35], and a method to monitor eating activities by monitoring changes in jaw movement with a device with an infrared sensor attached to the neck and irradiating light toward the submental area have been proposed [36]. However, because the device is worn, the design must be comfortable in daily life.

## 3. Proposed Method

This section describes the proposed method.

### 3.1. Basic Principle

The basic principle of the proposed method is as follows. This method uses infrared distance sensors and machine learning to recognize mouth movements during silent speech. When performing silent speech, the mouth moves, causing movements in facial skin areas, such as the cheeks and jaw joints. These skin movements are measured by multiple infrared distance sensors installed on eyewear or ear-mounted microphones. Infrared distance sensors measure the distance to a target point using infrared light. In the proposed method, infrared distance sensors measure changes in the distance between the sensors and the skin. A machine learning algorithm and pre-acquired training data are used to recognize silent speech actions from the time series data measured by multiple infrared distance sensors. The machine learning algorithm performs recognition based on the similarity of time series data, and we use Dynamic Time Warping (DTW) and Long Short-Term Memory (LSTM).

### 3.2. Prototype System

**Figure 1:**
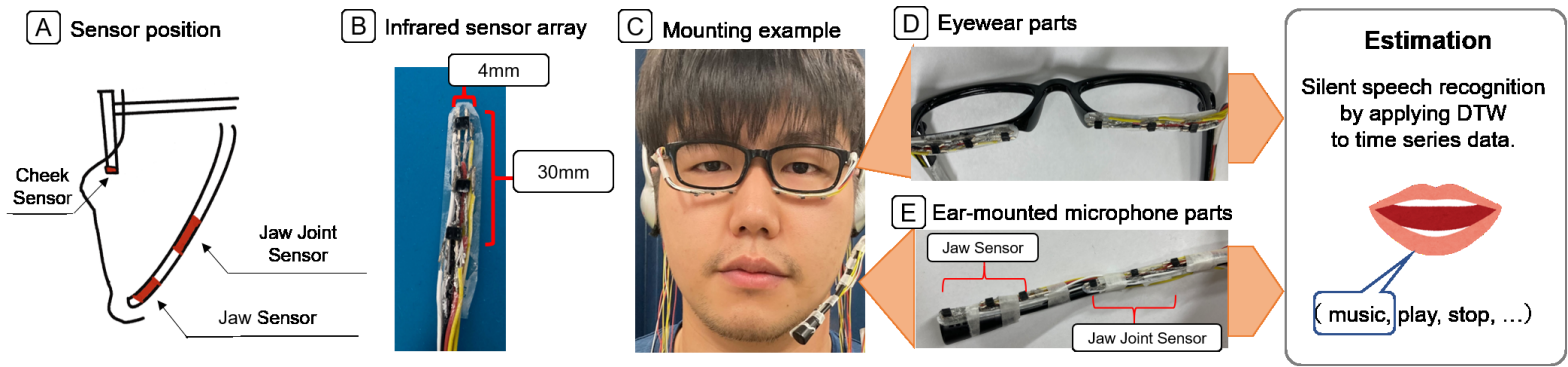
Prototype devices. Reprinted/adapted with permission from ref. [14] 2022, ACM.

We implemented our prototype system, with infrared distance sensors placed at the three positions shown in Figure 1A, to verify which position is most effective for applying the proposed method. We created sensor arrays consisting of three infrared distance sensors (TPR-105F, GENIXTEK CORP.) with a width of 4 mm and length of 30 mm, as shown in Figure 1B. Cheek movements were first sensed with six sensors (two arrays) placed on the lower rim of the glasses, as shown in Figure 1D. Jaw joint movements were then sensed with three sensors (one array) placed on the jaw joint side of the ear-mounted microphone, as shown in Figure 1E. Finally, jaw movements were sensed with three sensors (one array) placed on the jaw side of the ear-mounted microphone, as shown in Figure 1E. We used ear-mounted microphones since the proposed method was assumed to increase recognition accuracy by directly sensing mouth movements. Such a microphone component can be used as an attachment to an eyewear device or a stand-alone device.

### 3.3. Recognition Algorithm

DTW and a k-Nearest Neighbor (kNN) were used to recognize speech commands. Sensor values were normalized for each of the 12 sensors. The procedure is as follows. (1) DTW is used to calculate the similarity between the acquired data and training data. The training data include all speech-command data prepared in advance. The similarity is calculated for each sensor. (2) Data with high similarity to the acquired data are selected from the training data using the kNN. The affiliation probability of the acquired data is calculated from the proportion of speech-command labels of the selected data. The meaning of affiliation probability in this paper is the probability that indicates which speech command is the acquired data. For example, if the kNN (k = 3) selects three training data with speech-command label 1, the affiliation probability of speech-command label 1 is 1.0. This affiliation probability is calculated for each sensor. (3) The speech command label with the highest sum of the affiliation probabilities of all sensors is determined to be the recognition result of the acquired data. For example, if the total number of sensors is two and the affiliation probabilities of speech-command label 1 for sensors 1 and 2 are 0.3 and 0.4, respectively, the sum of the affiliation probabilities of speech-command label 1 for the acquired data is 0.7 (i.e., the total value for all sensors). LSTM was used instead of DTW from Evaluation 4 onwards. LSTM is a type of Recurrent Neural Network (RNN) used in machine learning and deep learning, particularly effective for processing time series and sequential data. While standard RNNs are designed to handle temporal dependencies in data, they often struggle with long-term dependencies. LSTM, with its unique structure, can effectively handle both short-term and long-term memory.

## 4. Evaluation 1. Short Speech

In Evaluation 1, we evaluated the recognition accuracy of the proposed method for five different speech commands. We also evaluated its recognition accuracy under three speech conditions (voiced speech, silent speech, and exaggerated silent speech). Ten male Japanese in their early 20s participated.

### 4.1. Speech Command

Five types of speech commands were used; “music”, “play”, “stop”, “next”, and “back” in Japanese. A previous study for simple hands-free input showed that recognizing about five types of commands is sufficient [37]. For the operation of a media player (e.g., music, video, still image), for example, these five types of commands are sufficient.

### 4.2. Procedures

Under the condition of exaggerated silent speech, the degree of mouth movement was instructed to be wider than the subject’s natural movement. The experimental task was to intentionally speak the commands displayed on the screen while wearing the sensor device and sitting on a chair. The five different speech commands were conducted ten times for each speech condition. Since one trial consisted of five commands, the next trial was conducted after finishing the previous trial instead of repeating the same command ten times. The order of the speech conditions was voiced speech, silent speech, and exaggerated silent speech. The acquired data for each subject consisted of 150 pieces (5 speech commands × 10 trials × 3 speech conditions). For accuracy evaluation, 10-fold cross-validation was conducted on the basis of intra-individual acquired data. The k in the kNN was set to 5 because that was the highest accuracy.

### 4.3. Results and Discussion

**Figure 2:**
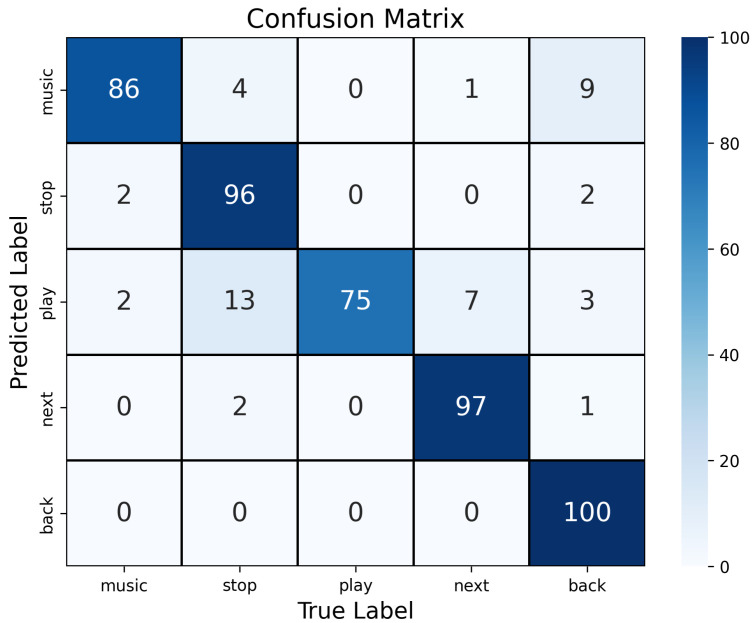
Confusion matrix of silent speech in Evaluation 1. Reprinted/adapted with permission from ref. [14] 2022, ACM.

Table 1 lists the results for each subject. Recognition accuracy decreased in the order of exaggerated silent speech, silent speech, and voiced speech with F-measure of 0.93 (min: 0.81, max: 1.00), 0.90 (min: 0.78, max: 1.00), and 0.86 (min: 0.64, max: 0.98), respectively. The confusion matrix of silent speech results is shown in Figure 2.

The results indicate the feasibility of the proposed method for simple hands-free input. In previous studies that examined simple hands-free input methods [26,37,38], it was shown that the methods recognized 5 to 7 types of input gestures of the face and eye gaze, with an F-measure ranging from 0.85 to 0.9. The proposed method has the same level of recognition accuracy as these methods and can be used as a simple hands-free input method. A previous study on the social acceptability of SSI [3] showed that SSI tends to be easily accepted by users because it is less conspicuous to others, even if it is less accurate than voiced interactions. Therefore, the accuracy of the proposed method is assumed to be socially acceptable.

The exaggerated silent speech had higher recognition accuracy than silent speech. The reason seems to be that the sensor values are characteristic of each speech command due to the wide mouth movement. Therefore, moving the mouth more widely is effective for the user to improve recognition accuracy.

## 5. Evaluation 2. Sensing Points

Although the prototype system measures three points (cheek, temporomandibular joint, and jaw), it would be useful if the proposed method could be used with fewer sensor points. Therefore, in Evaluation 2, we examined how much the recognition accuracy of the proposed method changes when the number of measurement points on the sensor device is reduced.

We compared four patterns: (1) one-point pattern (cheek) using eyewear, (2) two-point pattern 1 (jaw joint + jaw) using ear-mounted microphones, (3) two-point pattern 2 (cheek + jaw joint) using eyewear and ear-mounted microphones, (4) and three-point pattern (cheek + jaw joint + jaw) using eyewear and ear-mounted microphones. In this evaluation, we used the data of the silent speech for the five speech commands of the ten subjects in Evaluation 1 and conducted 10-fold cross-validation on the basis of intra-individual acquired data.

### Results and Discussion

Table 2 lists the results. (1) The one-point pattern (cheek) had an average F-measure of 0.78 (min: 0.64, max: 0.96). (2) Two-point pattern 1 (jaw joint + jaw) had an average F-measure of 0.86 (min: 0.74, max: 0.98). (3) Two-point pattern 2 (cheek + jaw joint) had an average F-measure of 0.87 (min: 0.73, max: 0.96). (4) The three-point pattern (cheek + jaw joint + jaw) had an average F-measure of 0.90 (min: 0.78, max: 1.00).

Since the one-point pattern (cheek) was less accurate than the other patterns, it would be more effective to add other parts to measure the jaw joint and jaw. However, this pattern is considered suitable if the user makes an effort to obtain enough accuracy, such as using exaggerated silent speech.

Since two-point pattern 1 (jaw joint + jaw) had sufficient accuracy in silent speech, the proposed method could be feasible with only the microphone. Since two-point pattern 2 (cheek + jaw joint) also had sufficient accuracy, the proposed method could be feasible without sensing the jaw if using eyewear to sense the cheek.

Although the three-point pattern (cheek + jaw joint + jaw) had the highest recognition accuracy, there was no significant difference from the two-point patterns. Therefore, reducing measurement points is appropriate if the two-point pattern has sufficient accuracy for the user’s purpose.

## 6. Evaluation 3. Longer Speech

In the previous evaluations, we evaluated relatively short speech. In Evaluation 3, we evaluated the proposed method for longer speech commands than in Evaluation 1. Ten Japanese male subjects in their early 20s participated. The experimental procedure was the same as in Evaluation 1, except the speech commands were changed, silent speech was used as the speech condition, and all sensors were used. We conducted 10-fold cross-validation on the basis of intra-individual acquired data.

### 6.1. Speech Command

Ten different speech commands were used for commands of voice assistants. The commands were (1) “Tell me the sports news”, (2) “Set a timer for 3 min”, (3) “What’s the weather tomorrow?”, (4) “Check the delivery status of my order”, (5) “What’s on your shopping list?”, (6) “Play a relaxing playlist”, (7) “Find a nearby convenience store”, (8) “What’s the title of this song?”, (9) “I want to go to the nearest station”, and (10) “Activate the line”. The speech was conducted in Japanese.

### 6.2. Results and Discussion

**Figure 3:**
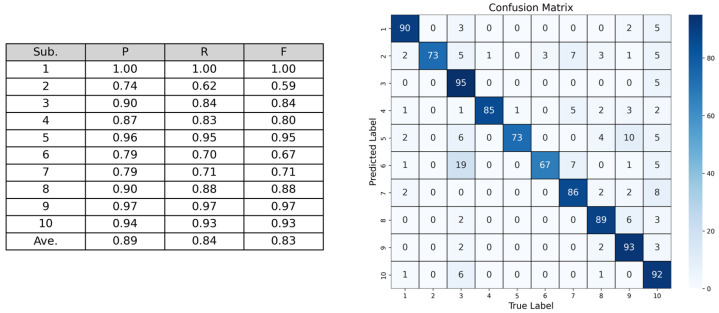
Recognition results of Evaluation 3. Reprinted/adapted with permission from ref. [14] 2022, ACM.

Figure 3 shows the results and confusion matrix. The average F-measure was 0.83. The results indicate the effectiveness of the proposed method for longer speech commands. The confusion matrix shows that most speech commands were recognized correctly. Recognition accuracy is assumed to increase by reducing the number of speech commands or the user moving his/her mouth widely, such as using exaggerated silent speech. We argue that the proposed method is feasible for use in voice assistants and other applications.

## 7. Evaluation 4. Many Commands and Using Deep Learning

This evaluation verified the following two points (1) Evaluation of the effectiveness of the proposed method when there are many speech commands: in Evaluation 1, the proposed method was evaluated for five types of speech commands. In contrast, in Evaluation 4, we evaluated the effectiveness of the proposed method for as many as 21 types of speech commands. (2) Evaluation of the accuracy of the proposed method when deep learning is used: in Evaluation 4, we evaluated whether the accuracy of the proposed method is improved by using deep learning LSTM compared with DTW and kNN used in Evaluation 1.

The subjects were 10 Japanese males in their early 20 s.

### 7.1. Speech Command

Twenty-one speech commands were prepared as shown in Table 3. These were selected from previous research [2] as those used to operate various digital devices.

### 7.2. Procedure

The experimental procedure was the same as in Evaluation 1, but the speech command was changed, and silent speech was used as the speech condition. All sensors were used. Recognition accuracy was obtained for three sensor point patterns of one sensor (cheek), two sensors (cheek, temporomandibular joint), and three sensors (cheek, temporomandibular joint, jaw). This was conducted to verify whether increasing the number of sensor points improves recognition accuracy when there are many speech commands. The differences between the two methods for machine learning were evaluated: one was the method using DTW and kNN (k = 5), which was used in previous evaluations, and the other was the method using LSTM, a deep learning analysis on time series data. The acquired data consisted of 210 samples (21 speech commands × 10 trials × 1 speech condition). Ten-fold cross-validation was performed and evaluated based on measurement data within individuals. From these, a total of six different results (three sensor point patterns × two machine learning patterns) were obtained.

LSTM was set up as follows. First, the input data were processed through a one-dimensional convolutional layer. The initial convolutional layer had 64 filters with a kernel size of 3, consisting of 2368 parameters. The subsequent convolutional layer also had 64 filters, adding 12,352 parameters. Following these layers, a 50% Dropout layer was applied to mitigate overfitting, along with a max-pooling layer that reduced the dimensionality of the feature map using a pool size of 2. Next, the output was flattened through a Time-Distributed Flatten layer. The entire sequence was then fed into an LSTM layer, which enables learning while considering long-term dependencies. After this, another Dropout layer was applied for additional overfitting prevention. The output layer consisted of a fully connected layer with 100 units, containing 10,100 parameters, and employed a ReLU activation function. Finally, a soft max-activated outputted layer generates the probability distribution.

### 7.3. Results and Discussion

**Figure 4:**
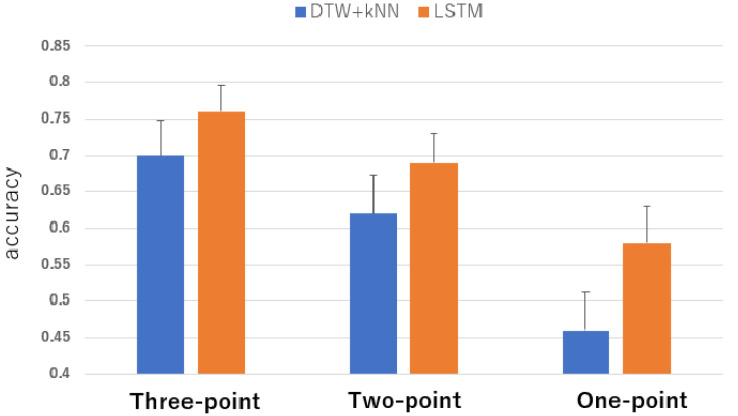
Percentage of correct answers for 21 speech commands according to different analytical methods between LSTM and a combination of DTW and kNN.

The overall results are shown in Figure 4. The results for each individual are shown in Table 4. The average accuracy rate of the measured data from the three points analyzed by DTW was 0.70, and the average accuracy rate analyzed by LSTM was 0.76. The average accuracy rate of the measurement data from two points analyzed by DTW was 0.62, and the average accuracy rate analyzed by LSTM was 0.69. The average accuracy rate of the measured data from one site analyzed by DTW was 0.46 and the average accuracy rate analyzed by LSTM was 0.58.

Effectiveness of the proposed method for cases with many speech commands: The results show that the proposed method can be used for cases with as many as 21 speech commands. This can be seen from the average accuracy rate of 0.76 when analyzed by LSTM using three sensor points. On the other hand, compared with the F value of more than 0.9 for the five types of speech commands in Evaluation 1, the results of this evaluation are not sufficiently accurate. Therefore, it is considered that when the number of speech commands is large, it is necessary to optimize the sensor position for each individual, as described in Evaluation 1.

The results also show that when the number of speech commands is large, the recognition accuracy improves as the number of sensor points increases. In Evaluation 1, there were five speech commands, so there was little improvement in recognition accuracy when the number of sensor points was increased from two to three, but it was found that a mechanism to increase the number of sensor points is effective when the number of commands is increased.

The effect of deep learning on accuracy: Overall, LSTM improved the accuracy rate more than DTW and kNN. For an individual example, Subject 8 showed a significant improvement, with a correct response rate of 0.57 for DTW and kNN, and 0.75 for LSTM. On the other hand, some subjects showed the opposite trend to the overall results. For example, Subject 1 showed a slight decrease in the rate of accuracy to 0.95 for DTW and 0.91 for LSTM. Overall, it was confirmed that the LSTM analysis increased the accuracy of this method. In addition, a comparison of the time required for analysis showed that the time required for 10-fold cross-validation of the measured data of one subject using DTW was about 10 min, whereas those using LSTM were about 1 min, thus significantly reducing the analysis time.

## 8. Evaluation 5: Walking and Re-Attachment of the Device

The measurement data used in the previous evaluations were measured while sitting on a chair after wearing the device once, but when the device is actually used as a wearable device, it is assumed that the device is put on and taken off and used while walking. Therefore, in Evaluation 5, the accuracy of the proposed method was evaluated when the device was re-attached and when walking. The subjects were five Japanese males in their early twenties.

### 8.1. Experimental Conditions

Silent speech was employed. The following three experimental conditions were used. (1) Stationary condition: This condition was the same as in previous experiments. This was a condition in which the speech command was given while the subject was seated in the chair. (2) Re-attachment condition: This condition was the same as in previous experiments. This was a condition in which the speech command was given while the subject was seated in the chair. (3) Walking condition: This was a condition in which the speech command was given while walking. Walking (3 km/h) was performed on a treadmill.

### 8.2. Procedure

In each experimental condition, 21 different speech commands were given 10 times each. The same speech command was not performed consecutively. One trial of each of the 21 speech commands was followed by the next trial. The acquired data consisted of 630 samples (21 speech commands × 10 trials × 3 speech conditions). LSTM, which was confirmed to be effective in Evaluation 4, was used for machine learning.

The machine learning training data were divided into the following two analysis patterns: (1) Patterns that only use data within the same conditions: In these patterns, 10-fold cross-validation was carried out on data within the same conditions. (2) A pattern that uses data from the stationary condition as training data: In this pattern, for the stationary condition, 10-fold cross-validation was carried out using only the data within the stationary condition. For the re-attachment and walking conditions, the data from the stationary condition was used as training data for 10-fold cross-validation. The speech commands were evaluated in the following two categories: (1) 5 patterns and (2) 21 patterns. From these, a total of 12 types of results (2 patterns of evaluation data × 3 patterns of training data × 2 patterns of speech commands) were obtained.

### 8.3. Results and Discussion

**Figure 5:**
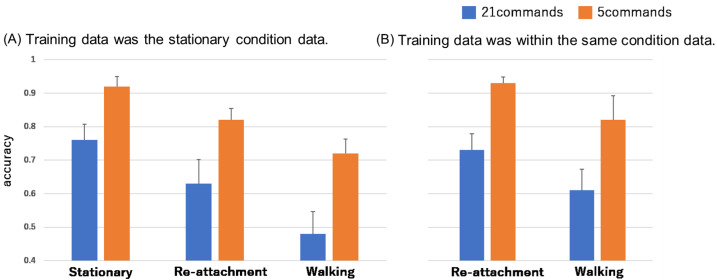
Rate of accuracy for each experimental condition when data training data were the stationary condition data or the same experimental condition data.

The rate of accuracy for each experimental condition is shown when the data from the stationary condition is used as training data. The overall average is shown in Figure 5. The results for each individual are shown in Table 5. In the stationary condition, the average accuracy rate was 0.76 for the 21 speech commands and 0.92 for the 5 speech commands. In the re-attachment condition, the average accuracy rate was 0.63 for the 21 speech commands and 0.82 for the 5 speech commands. In the walking condition, the average accuracy rate was 0.48 for the 21 speech commands and 0.72 for the 5 speech commands.

Next, the rate of accuracy for each experimental condition is shown when data within the same experimental condition is used as training data. In the re-attachment condition, the average accuracy rate was 0.73 for the 21 speech commands and 0.93 for the 5 speech commands. In the walking condition, the average accuracy rate was 0.61 for the 21 speech commands and 0.82 for the 5 speech commands

For the re-attachment condition: Analysis of the measurement data for the re-attachment condition based on the training data for the stationary condition showed that the average accuracy rate decreased. This is considered to be due to a shift in the measurement point at the time of re-attachment. Therefore, it is necessary to ensure that the measurement point does not shift with each re-attachment. On the other hand, the average accuracy rate of re-attachment based on the training data of the re-attachment condition was not so decreased from those in the stationary condition. Therefore, it is considered that the proposed method can be used with high accuracy if the training data is re-collected at the time of re-attachment.

Regarding the walking condition: When using training data from the stationary condition, the recognition accuracy for test data in the walking condition was lower compared with other conditions. This is likely because, in the walking condition, body vibrations cause shifts in the infrared distance sensor’s measurement position, resulting in lower similarity between the stationary and walking data. On the other hand, when using training data from the walking condition, the average recognition accuracy for the five command types of the walking condition was 0.82, and four out of five subjects (excluding Subject 3, who had significantly lower accuracy) had an accuracy ranging from 0.86 to 0.94. These results indicate that the proposed method can be applied in the walking condition if limited to around five commands by utilizing walking condition training data. However, obtaining training data in the walking condition requires a little effort for users.

## 9. Limitations and Future Works

### 9.1. Individual Difference That May Influence the Recognition Accuracy

The accuracy of the method we propose may be affected by several factors. Future work will involve testing these factors with subjects possessing a range of characteristics. (1) In this study, the number and attributes of the participants were limited. Since the participants were primarily young Asians, we plan to evaluate individuals with a more diverse range of attributes in the future. Regarding the number of participants, we believe that we have obtained sufficient data to discuss the evaluation content. However, we intend to increase the number of participants to investigate the identification of individuals for whom the proposed method is less effective and to examine the degree of generalization. (2) The color of the skin. The method’s recognition accuracy might be less for individuals with extremely dark skin, as infrared distance sensors often struggle to respond to the color black. (3) Any obstruction that comes between the sensor’s front and the skin could potentially lower the method’s recognition accuracy. For instance, the presence of a hair bundle from a user with long hair or a large beard in front of the sensor might decrease the recognition accuracy.

### 9.2. Other Factors That May Influence Recognition Accuracy

The method we propose can maintain accuracy equivalent to the results presented in this paper, provided that the device is positioned approximately the same. However, if the device’s attachment point significantly shifts upon reattachment, it is plausible that the recognition accuracy will diminish. In such scenarios, it is advisable to reapply the proposed method by recollecting the gesture training data. Given that the gesture training data can be gathered swiftly, it is reasonable to assume that the user’s burden is minimal. In the case of eyeglass-type devices, misalignment of the wearing position affects the accuracy, so functions that enable the device to be worn in the correct position and that recognize misalignment and correct the data are considered necessary. This paper did not investigate the scenario of mounting position misalignment, making this verification a subject for future research.

We plan to evaluate the proposed method in environments with different lighting intensities. One factor impacting the accuracy of our proposed method is the ambient light’s intensity. This influence stems from the fact that the readings of the infrared distance sensor exhibit variations in outdoor and indoor settings due to the differing levels of ambient light in these environments. While consistent infrared distance sensor readings can be achieved under uniform lighting conditions, it is imperative to investigate whether it is essential to undergo a retraining process for our system when transitioning into an environment characterized by a distinct lighting intensity. Furthermore, it is worth noting that our evaluation will also consider factors such as dynamic lighting changes that occur throughout the day, as they can impact the performance and adaptability of the proposed method in real-world scenarios.

Additionally, we plan to survey the user’s comfort and ease of use with the device. If the device causes discomfort or is difficult to use, it may impact the user’s willingness to use it consistently, which could in turn affect the recognition accuracy. This aspect could also be considered in future studies to enhance the usability and effectiveness of the proposed method.

In this paper, we investigated the recognition accuracy by having the subjects speak a predefined speech content, but we believe that natural language recognition is necessary to apply the proposed method in various real-world situations, so this verification will be the subject of future research.

In our proposed method, machine learning was performed on the data of each individual due to the fluctuation of data caused by the facial features of each individual, but by creating a group of users with similar data, there is a possibility of increasing the amount of data, improving recognition accuracy and reducing the cost of machine learning such as data collection and learning time. This is a subject for future research.

Note that this paper focuses solely on recognition performance and does not consider real-time recognition performance or data processing. This is also a future challenge, and it aligns with other similar papers.

In this paper, we evaluated the recognition accuracy of speech commands using intra-user data, so users would need to acquire their own training data when using the proposed system. It is not a very tedious task, as the system can be utilized by collecting about 10 trials per voice command (which takes a few dozen seconds). If the proposed method could utilize other users’ training data, it would eliminate the need for users to collect their own data, which would be advantageous. However, this aspect has not been achieved in this paper, and we consider it a future challenge.

## 10. Conclusions

In this study, we investigated a method to recognize speech content using a spectacle-type device with an infrared distance sensor to investigate the possibility of silent speech interaction by a wearable device that can be easily worn. The proposed method estimates the speech content by measuring the facial skin movements that occur with a speech from infrared distance sensors installed on the lower rim of the glasses and on the cheek and chin sides of the ear-mounted microphone. A prototype device was created to realize the proposed method. In Evaluation 1, five speech commands were selected for the media player, and the recognition accuracy was evaluated using three different speech methods: voiced speech, silent speech, and exaggerated silent speech. The results show that the method can provide the function of silent speech interaction with spectacles. It was also found that the recognition accuracy could be improved by the user’s effort. In Evaluation 2, we evaluated whether the recognition accuracy changes with the combination of sensor points. The results show that the recognition accuracy decreased when the number of sensor points was reduced and that attachment parts were effective in this method. In Evaluation 3, the recognition accuracy of ten long speech commands was evaluated using silent speech. The results show that the method can be used in voice assistants. In Evaluation 4, we evaluated the case with many speech commands and the case using deep learning. The results show that the method is effective even when there are many speech commands. In addition, when deep learning was used, the accuracy was improved and the time required for analysis was significantly reduced. In Evaluation 5, the recognition accuracy was evaluated when walking and when the device was re-attached. The results show that the recognition accuracy could be reduced by analyzing data within the same conditions. These results show that the proposed method can be implemented as a simple hands-free input interface.

In the evaluation of this paper, it was found that the accuracy of the measurement with the prototype device varied depending on the facial features of each individual, so in the future, we will create a device that allows fine adjustment of the sensor installation position and angle so that the device can be worn according to the user’s facial features, and verify whether the recognition accuracy can be improved. We will verify whether the recognition accuracy improves. We have also found that accuracy is reduced by device misalignment, so we will verify whether the recognition accuracy can be improved by creating a device that can be worn without moving.

## Figures and Tables

**Table 1:** Recognition results for each subject (R: recall, P: precision, F: F-measure). Reprinted/adapted with permission from ref. [14] 2022, ACM.

Sub.	Voiced Speech			Silent Speech			Exaggerated Silent		
	P	R	F	P	R	F	P	R	F
1	0.98	0.98	0.98	0.98	0.98	0.98	1.00	1.00	1.00
2	0.85	0.84	0.83	0.84	0.80	0.78	0.91	0.90	0.90
3	0.94	0.92	0.91	0.89	0.86	0.86	0.95	0.94	0.97
4	0.94	0.92	0.92	0.95	0.94	0.94	0.85	0.82	0.81
5	0.92	0.92	0.92	1.00	1.00	1.00	1.00	1.00	1.00
6	0.88	0.88	0.88	0.95	0.94	0.94	0.92	0.90	0.90
7	0.72	0.68	0.64	0.89	0.86	0.85	0.91	0.90	0.90
8	0.66	0.66	0.65	0.89	0.84	0.84	0.92	0.90	0.90
9	0.94	0.92	0.92	0.95	0.94	0.94	0.98	0.98	0.98
10	0.91	0.90	0.90	0.93	0.92	0.92	0.95	0.94	0.94
Ave.	0.87	0.86	0.86	0.93	0.91	0.90	0.94	0.93	0.93

**Table 2:** Recognition results for each measurement point. Reprinted/adapted with permission from ref. [14] 2022, ACM.

	(1) One-Point			(2) Two-Point Pattern 1			(3) Two-Point Pattern 2			(4) Three-Point		
	P	R	F	P	R	F	P	R	F	P	R	F
Ave.	0.81	0.79	0.78	0.89	0.87	0.86	0.90	0.88	0.87	0.93	0.91	0.90
max	0.99	0.99	0.99	1.00	1.00	1.00	1.00	1.00	1.00	1.00	1.00	1.00
min	0.57	0.59	0.57	0.70	0.67	0.63	0.73	0.71	0.68	0.75	0.73	0.70
SD	0.18	0.15	0.15	0.15	0.15	0.16	0.13	0.14	0.15	0.13	0.13	0.14

**Table 3:** Twenty-one types of speech commands.

music	cancel	answer	play	next	left
yes	menu	Alexa	back	ok	right
no	open	mute	start	stop	
home	close	play music	stop music		

**Table 4:** Rate of the accuracy for 21 speech commands for each individual according to different analytical methods between LSTM and a combination of DTW and kNN.

	DTW + kNN			LSTM		
Sub.	Three	Two	One	Three	Two	One
1	0.95	0.88	0.63	0.91	0.87	0.73
2	0.54	0.48	0.43	0.64	0.60	0.58
3	0.52	0.42	0.25	0.73	0.62	0.48
4	0.68	0.63	0.42	0.75	0.65	0.55
5	0.90	0.84	0.54	0.91	0.89	0.82
6	0.65	0.55	0.26	0.65	0.61	0.38
7	0.59	0.49	0.34	0.59	0.53	0.39
8	0.57	0.47	0.35	0.75	0.63	0.49
9	0.81	0.75	0.67	0.88	0.84	0.79
10	0.75	0.68	0.65	0.75	0.63	0.63
Ave.	0.70	0.62	0.46	0.76	0.69	0.58

**Table 5:** Accuracy of commands for each experimental condition.

	(A) Training Data Is the Stationary Condition						(B) Training Data Is the Same Condition			
	Stationary		Re-Attachement		Walking		Re-Attachement		Walking	
Sub.	21	5	21	5	21	5	21	5	21	5
1	0.82	0.98	0.64	0.82	0.54	0.83	0.71	0.90	0.61	0.86
2	0.91	0.96	0.84	0.90	0.63	0.78	0.86	0.98	0.72	0.90
3	0.63	0.82	0.39	0.70	0.23	0.58	0.56	0.94	0.38	0.54
4	0.73	0.88	0.63	0.88	0.49	0.73	0.75	0.88	0.61	0.88
5	0.70	0.96	0.66	0.82	0.51	0.68	0.74	0.96	0.73	0.94
Ave.	0.76	0.92	0.63	0.82	0.48	0.72	0.73	0.93	0.61	0.82

## Data Availability

Data are contained within the article.
